# Influenza Vaccination Strategies Should Target Children

**DOI:** 10.1093/phe/phx021

**Published:** 2017-12-08

**Authors:** Ben Bambery, Thomas Douglas, Michael J Selgelid, Hannah Maslen, Alberto Giubilini, Andrew J Pollard, Julian Savulescu

**Affiliations:** 1 Monash University; 2 University of Oxford

## Abstract

Strategies to increase influenza vaccination rates have typically targeted healthcare professionals (HCPs) and individuals in various high-risk groups such as the elderly. We argue that they should (instead or as well) focus on increasing vaccination rates in children. Because children suffer higher influenza incidence rates than any other demographic group, and are major drivers of seasonal influenza epidemics, we argue that influenza vaccination strategies that serve to increase uptake rates in children are likely to be more effective in reducing influenza-related morbidity and mortality than those targeting HCPs or the elderly. This is true even though influenza-related morbidity and mortality amongst children are low, except in the very young. Further, we argue that there are no decisive reasons to suppose that children-focused strategies are less ethically acceptable than elderly or HCP-focused strategies.

## Introduction

Though many consider it to be a trivial and self-limiting viral illness, influenza can cause serious illness or even death (CDC, 2016a). It has been estimated that influenza kills between 250,000 and 500,000 people each year, making it one of the most deadly infectious diseases affecting humans (WHO, 2014). The virus most severely affects infants and children under 2 years of age (WHO, 2014) and the very old. Approximately 90 per cent of influenza-related deaths occur in individuals over age 65 years (CDC, 2016b). Erosion of the immune system during old age leads to a predisposition to serious complications of influenza infection, such as pneumonia, as well as a poor immune response to the influenza vaccine (Reichert *et al.*, 2004). Thus, even elderly people vaccinated against influenza may die as a result of influenza infection.

While influenza prevention efforts have traditionally targeted vaccination of HCPs, the elderly and others particularly vulnerable to infection (e.g. pregnant women, those with chronic respiratory diseases), this strategy has not consistently decreased influenza-related mortality rates, and the most positive findings are open to dispute. Though ecological evidence previously suggested reductions in all-cause mortality up to 50 per cent amongst vaccinated elderly persons, this figure has been shown to be afflicted by bias and some now consider it inaccurate (Jackson *et al.*, 2006a, 2006b). Researchers have shown that vaccination uptake amongst elderly citizens in the USA increased from 31 per cent in 1989 to 66 per cent in 1997; yet, all-cause influenza mortality outcomes increased during this time period, even correcting for population ageing and the sub-type of circulating viral strain (Simonsen *et al.*, 2005). More recent trends from the the Centers for Disease Control and Prevention (CDC) in the USA continue to suggest an increase in influenza-related death rates over the past decade (CDC, 2010). This parallels stable or increasing mortality trends in Spain and Austria, where good data are also available (Kuo *et al.*, 2011, López-Cuadrado *et al.*, 2012). In the UK, modestly declining mortality rates have been reported in recent decades, despite consistently high vaccination uptake (70 per cent coverage) amongst elderly citizens (Matias *et al.*, 2016).

While growing and aging populations may partly explain trends of stable or increased mortality, it is clear that the goals of influenza prevention strategies (e.g. to decrease serious morbidity and mortality from influenza) are not currently being achieved in many industrialized nations. In a blogpost published in the *American Journal of Bioethics* in February 2014, Arthur Caplan and Dorit Reiss briefly addressed the implications of a policy requiring children aged between 6 and 59 months to be vaccinated against influenza before entering pre-school or day-care programmes. This policy had been proposed by the Public Health Department of Rhode Island (Caplan and Reiss, 2014). Some of the present authors had previously published a commentary (reference removed for blind review) in which it was briefly argued that the most efficient strategy for decreasing influenza-related mortality might be mandatory vaccination of children.

In this article, we further strengthen our epidemiological and ethical arguments for vaccinating children against influenza, though here we will not argue specifically for mandatory vaccination—our arguments instead apply to all programmes for increasing vaccination rates. For example, they apply to programmes that seek to increase vaccination rates by requiring not that individuals actually undergo vaccination, but merely that they report their vaccination status (Dare, 1998). Our primary goal is to consider who should be the target of pro-vaccination strategies, regardless of what precise form those strategies take. We will do this by examining the benefits and burdens of vaccinating children compared to the elderly or HCPs.

Our argument proceeds in three steps. We first argue that vaccination strategies targeted at children are likely to be more effective at reducing overall influenza morbidity and mortality than comparable strategies targeting HCPs or the elderly. This conclusion creates a presumption, from a public health standpoint, in favour of supplementing or replacing existing strategies with child-focused strategies. We then weigh the potential moral costs of vaccination strategies targeting children by considering three possible ethical objections to child-focused strategies, arguing that none of these is decisive: the moral costs associated with child-focused strategies do not outweigh the public health benefits. Finally, we explore the practicalities of pro-vaccination strategies targeting children, concluding that increasing vaccination uptake could be cost-effective and facilitated annually through school-based ‘outreach’ vaccination programmes, such as the current programme in the UK.

## Part 1—Effectiveness in Reducing Total Influenza Burden

In this section we evaluate and compare child-, HCP- and elderly focused vaccination strategies with respect to their effectiveness in reducing the total health costs (morbidity and mortality) of influenza and influenza vaccination. We will henceforth sometimes refer to these costs as the ‘total burden’ of influenza. We deploy the following desiderata to appraise a given pro-vaccination strategy in reducing this burden: (i) reduction in influenza-related morbidity and mortality among the vaccinated group, (ii) acceptable safety of the vaccine for the vaccinated group and (iii) reduction in influenza-related morbidity and mortality for others.

Table 1 summarizes the performance of three different vaccination strategies against these desiderata. Vaccination uptake rates are in reference to both the USA and the UK, where uptake rates are easily accessible online and important differences exist in vaccine delivery for children. On the one hand, in the USA, vaccines are officially recommended for all children aged >6 months, who may receive vaccination through their primary care doctor at an out of pocket cost. On the other hand, in the UK a school-based influenza vaccination programme has been in effect since 2012, where primary school-aged children are offered the influenza vaccine for free during school hours at school. As of the 2016/2017 influenza season, the vaccine is currently offered to all healthy English children aged between 2 and 7 years, with some ‘pilot’ areas of the country offering vaccination to all children aged between 2 and 11 years (England, 2017). We will reference vaccine uptake only in areas where flu vaccination is offered as part of this programme (e.g. those areas where the vaccine is free and offered in school).

**Table 1.** Comparison of vaccinating the elderly, HCP and children against influenza using recent vaccination uptake rates from the USA and UK

	Elderly		HCP		Children	
Vaccination uptake	The UK 70 per cent	The USA 66 per cent	The UK 51 per cent	The USA 77 per cent	The UK 30–63 per cent^3^	The USA 59 per cent^4^
Vaccination in medical interests?	✓		✓		✓	
Potential for reducing total influenza-related deaths?	**Limited**		**Limited**		**Great**	
VE (per cent)	23–50		88–89		79–87	
Case-fatality rate for unvaccinated	?		?		>1/million	
*Case-fatality rate for vaccinated*	<1/33 million		<1/33 million		<1/25 million	
Risks of vaccine:						
Common	Injection site pain		Injection site pain		Injection site pain, wheeze (only with LAIV; no significant common risk with the trivalent influenza vaccine), headache	
Severe	Immediate hypersensitivity (1/million)		Immediate hypersensitivity (1/million)		Hospitalizations or life-threatening allergy (1/5 million)	
	GBS (2/million at most, some studies found no correlation)		GBS (2/million at most, some studies found no correlation)			

Statistics for demographic-specific influenza mortality are relevant to our analysis of whether the influenza vaccine is in the medical interests of individuals in the different groups (for example, to compare the chance of being harmed or dying from the flu when unvaccinated versus being harmed or dying from receiving the vaccine). We have taken these figures from the CDC’s website (CDC, 2010, 2016a, 2016b), which provides age-related mortality data that can easily be compared to vaccine uptake rates. It can be expected that these statistics would roughly generalize to other countries, as typical, healthy individuals in each group should not behave, immunologically speaking, significantly different from ‘matched’ individuals from other countries. For example, the average healthy American child would not be expected to suffer greater complications from contracting influenza than the average British child, nor be at higher risk of an allergic reaction from the vaccine, just because he or she is American.^1^ Calculated case-fatality rates should therefore be transferrable between both countries, and not limited to the USA, where the data were obtained.

Estimates regarding the effectiveness of the vaccine to prevent influenza and the risks posed by vaccination were taken directly from the Cochrane Library (Jefferson *et al.*, 2010a, 2010b; Jefferson *et al.*, 2012; Demicheli *et al.*, 2014) and, where necessary, supplemented by a PubMed search limited to randomized control trials conducted in the past 20 years. To minimize bias, studies were only considered if they included more than 200 participants.

It is difficult to quantify and directly compare decreases in total influenza-related mortality between the three groups (children, HCPs and the elderly), and various models are used in different countries to gauge influenza-related mortality seasonally. For example, the CDC currently uses respiratory and circulatory (R&C) deaths recorded on hospital death certificates to estimate influenza-related mortality in the USA (CDC, 2016b). This measurement is not sensitive to influenza-related deaths not recognized or recorded in hospital settings, for example people who die from secondary complications of influenza infection like bacterial pneumonia, or who die before the virus can be detected in a laboratory (Bisno *et al.*, 1971; Douglas, 1976). This measure may thus underestimate the true burden of disease.

Complex statistical methods are sometimes used to estimate the number of deaths above a specified threshold that occur in winter, which is a technique used in the UK, allowing researchers to then correlate these with influenza surveillance data to estimate the mortality attributable to influenza in a given year (Dushoff *et al.*, 2006). Though this method may account for deaths not recorded or recognized on hospital death certificates, it assumes the pattern of non-influenza seasonal mortality remains constant year by year, which may not be the case. Again, then, this is not a perfect measure.

In light of the limitations and complexities in accurately measuring influenza-related death rates, and thus the difficulty in quantifying and comparing this between children, HCP and the elderly, we simply define prevention strategies that are likely to decrease either R&C deaths or all-cause deaths by 25 per cent or less as having a ‘limited’ effect on decreasing total influenza-related mortality, between 25 and 50 per cent as having a ‘moderate’ effect, and greater than 50 per cent as having a ‘great’ effect. This will allow sufficient comparison for the purposes of this article.

## Vaccinating the Elderly

In total, 66 per cent of elderly US citizens were vaccinated against influenza during the 2014/2015 influenza season (CDC, 2016a). Vaccination coverage has been in excess of 63 per cent each year since 1997 with the exception of one season (where coverage was 60 per cent) (CDC, 2016c). Vaccination rates are similar in other countries like the UK, where on average 70 per cent of elderly citizens were vaccinated between 2001 and 2011 (OECD, 2016).

### What Are the Benefits of the Vaccine for Vaccinated Individuals?

Because influenza vaccination has long been established as best practice in the prevention of influenza for elderly adults, traditional study structures and methodology necessary to meet requirements for inclusion in a Cochrane analysis such as placebo-controlled trials are generally considered unethical. Consequently, there is little good quality evidence regarding the protective efficacy of the influenza vaccine amongst the elderly (Jefferson *et al.*, 2010a).

A randomized controlled trial (RCT) conducted in 1994 in The Netherlands provides the best quality evidence available. Researchers assessed the protective effect of the influenza vaccine against culture-confirmed influenza in 1838 elderly adults who were >60 years old. It was found that those vaccinated against influenza (the experimental group) were half as likely to be diagnosed with influenza as those unvaccinated (the control group), suggesting a vaccine efficacy (VE)—i.e. the reduction in disease incidence in a vaccinated group compared to an unvaccinated one—of 50 per cent against culture-confirmed influenza for adults >60 years old (Govaert *et al.*, 1994). However, stratification by age showed a steep decline of VE with age, where those >70 years old conferred only 23 per cent protection against influenza infection.

### What Are the Health Risks of the Vaccine for Vaccinated Individuals?

The Cochrane Review suggests the safety profile of the influenza vaccine is acceptable for elderly adults independent of any potential benefits gained from vaccination (Jefferson *et al.*, 2010a). The CDC estimate that injection site pain is the most common side effect associated with the inactivated influenza vaccine, which is the only licenced vaccine available for elderly adults. Injection site pain affects up to 65 per cent of those vaccinated and usually resolves within 2 days without treatment (CDC, 2016d).

As for more serious adverse effects, the risk of immediate hypersensitivity (or severe allergy) following influenza vaccination is recognized by the CDC (CDC, 2016d). The risk of immediate hypersensitivity is usually considered to be around 1.5 in 1 million (from any vaccine) (Fiore *et al.*, 2010). The risk of developing Guillain–Barré syndrome (GBS)—a neurological condition that may result in long-term nerve damage and death—is considered to be at most around 1–2 per million influenza vaccines given to adults (CDC, 2016d), although some studies, and particularly those published after the 1970s, found no increased risk of GBS associated with influenza vaccines (Haber *et al.*, 2009). Though most individuals with GBS recover fully, in rare cases people can die (CDC, 2015a). The mortality rate of GBS can be as high as 2.8 per cent in the first 6 months and 3.9 per cent in the first year (Van den Berg *et al.*, 2013). Using these figures, we can extrapolate that the case-fatality rate for the influenza vaccine is therefore likely to be very low and likely <1 in 33 million in the elderly.

### What Are the Benefits of the Vaccine for Non-vaccinated Individuals?

There is no good direct evidence on the wider benefits of vaccinating elderly people. However, it appears that substantial increases in vaccination uptake by elderly populations in recent years have not significantly decreased total influenza-related deaths in several countries. Simonsen *et al.* (2005) have shown that increases in vaccination uptake in the USA—from 31 per cent in 1989 to 66 per cent in 1997—were not associated with decreases in influenza-associated deaths but with increases in excess mortality and influenza-associated hospitalization rates (Simonsen *et al.*, 2005), while more recently CDC death records and all-cause mortality estimates show that influenza-related mortality has increased substantially (by a figure of thousands) over the past few decades, despite improvements in uptake (Thompson *et al.*, 2003; CDC, 2010), although the increase might be explained, at least in part, by improved diagnostics. Similar stability or increase in influenza-related mortality has also been observed over a 6-year period in Spain, despite increases in vaccine uptake from 60 to 70 per cent from 1999 to 2005 (López-Cuadrado *et al.*, 2012).

We therefore quantify the potential reductions in total influenza-related mortality by vaccinating the elderly as ‘limited’ according to our criteria (i.e. likely <25 per cent).

## Vaccinating HCP

In theory, vaccinating HCP against influenza may prevent the transmission of harmful influenza infections between HCP and patients (Poland *et al.*, 2005). Government initiatives in the USA and UK (amongst other countries) have launched various campaigns in recent years attempting to increase vaccination uptake through educating HCP about the benefits of vaccination and increasing access within the workplace (Babcock *et al.*, 2010) while also including HCP in official influenza vaccine recommendations nationally.

The US Public Health Service’s draft Healthy People 2020 has set a target of 90 per cent vaccination coverage for HCP (National Vaccine Advisory Committee, 2013), but estimates taken from the CDC show coverage rates of only 77 per cent during the 2015/2016 influenza season (CDC, 2015b). Rates during the 2015/2016 season in the UK were even lower, where 50.6 per cent of frontline healthcare workers were reported to have had the influenza vaccine (England, 2016).

### What Are the Benefits of the Vaccine for Vaccinated Individuals?

Similar to the elderly, there is not enough strong evidence available for the Cochrane Review to draw any conclusion regarding effectiveness of HCP vaccination. The Cochrane Review estimates that around 71 healthy adults need to be vaccinated to prevent one set of influenza symptoms (Demicheli *et al.*, 2014).

An RCT conducted in the US state of Maryland over 3 consecutive years from 1992 through to 1995 provides some assessment of the effectiveness of HCP vaccination: researchers found that HCP vaccinated against influenza had 88 per cent fewer cases of influenza A infection and 89 per cent fewer cases of influenza B infections (Wilde *et al.*, 1999). The sample size was, however, small (*n* = 264).

As for other benefits, there is some evidence suggesting vaccination of HCP decreases work absenteeism (by as much as 28 per cent according to some studies) (Saxen and Virtanen, 1999), though the Cochrane reviewer believes decreases in work absenteeism are likely to be ‘limited and minimal’ (Jefferson *et al.*, 2010b).

### What Are the Risks of the Vaccine for Vaccinated Individuals?

The risks of vaccination for healthy adults are estimated to be similar to the risks for elderly citizens as examined above: the most common side effect of the influenza vaccine for adults is injection site pain; the most serious risks are the development of severe allergy (1 per million) and possibly GBS (at most 1–2 per million, but as noted above some studies found no correlation between vaccination and GBS); and the case-fatality rate for HCP vaccinated against influenza would also be very low and certainly less than 1 in 33 million (which is probably also an overestimate).

### What Are the Benefits to Unvaccinated Individuals?

Strong evidence from long-term facilities (e.g. nursing homes) shows that increased uptake of vaccination by HCP may decrease influenza rates, morbidity and mortality (Johnson and Talbot, 2011). In contrast, a systematic review published in 2006 recorded inconclusive findings on the effect of staff vaccination and rates of influenza amongst elderly patients in acute care facilities such as hospitals (Thomas *et al.*, 2006). More recent (albeit smaller) studies have shown some improvement in patient outcomes when staff are vaccinated against influenza, but the results of these have been undermined by relatively low levels of vaccination uptake amongst participants (Carman *et al.*, 2000). In general, one would expect HCP vaccination to have a smaller effect on morbidity and mortality in acute care settings than in long-term facilities, where contact with HCP is ongoing and patients generally suffer more comorbidities.

Overall, fewer than 10 per cent of elderly citizens (those who may be the most at risk) in the USA reside within healthcare institutions (U.S. Census Bureau, 2014: 136), while in the UK it is fewer than 5 per cent (Officer For National Statistics, 2013). This suggests that vaccinating HCP against influenza will protect only a small proportion of elderly citizens—the majority live in the community. We therefore quantify the potential reductions in total influenza-related mortality by vaccinating HCP as ‘limited’ according to our criteria (e.g. likely <25 per cent).

## Vaccinating Children

Because naïve immune systems respond less effectively, children are more likely than adults to become sick from influenza infection, and to remain sick for longer periods of time. Due to lower pre-existing immunity, children's viral load is higher than adults’, and the period during which children can actively transmit infections to others is longer, thus increasing spread of disease. Studies have shown that children frequently introduce influenza into households and that schools, in particular, act as conduits for disease transmission (Glezen, 2006).

The frequency with which children suffer influenza illness, and the ease with which they pass infection on to others, has driven many research teams to study the potential advantages of vaccinating children to promote herd protection against influenza. ‘Herd protection’ here refers to the indirect protection offered to unvaccinated individuals when a sufficient proportion of a population is vaccinated against a disease. Because childhood transmission is a major driver of annual influenza epidemics, increasing vaccination uptake among children may therefore limit the widespread dissemination of infection into the community (John and Samuel, 2000). This would be important for decreasing the total burden of seasonal epidemics and reducing exposure of vulnerable groups (e.g. the elderly, those with chronic lung diseases) for whom the vaccine may not confer adequate protection. A plethora of convincing evidence demonstrates this effect (Monto *et al.*, 1970; Reichert *et al.*, 2001; Reichert, 2002; Glezen, 2006; Glezen and Simonsen, 2006; Basta *et al.*, 2009; Longini, 2012; Pebody *et al.*, 2016).

In 2008 the Advisory Committee on Immunization Practices (a sub-section of the CDC) first began recommending annual influenza vaccination for all US children aged 6 months to 18 years (Fiore *et al.*, 2008). Vaccination coverage for US children aged between 6 months and 17 years in the 2015/2016 season was 59.3 per cent, up from 43.7 per cent during the 2009/2010 season, suggesting some response to these recommendations (CDC, 2016a). In the UK, all children aged between 2 and 7 years are currently offered the vaccine free as part of school-based influenza immunization programmes, with an ongoing roll out planned to age 11 years and some areas of the country already offering free vaccination at school to children aged up to 11 years. In areas of the UK with programmes extending to 11 years of age, 60.3 per cent of children aged 5–10 years and 63 per cent aged 10–11 years received vaccination in 2016/2017 season, while only 35.4 per cent of 2-year olds, 37.7 per cent of 3-year olds and 30 per cent of 4-year olds were vaccinated in the same season, in primary care (England, 2017).

### What Are the Benefits of the Vaccine for Vaccinated Individuals?

The Cochrane review estimates that 28 children over the age of 6 years need to be vaccinated to prevent one case of influenza (infection and symptoms) and 6 children under the age of 6 years need to be vaccinated to prevent one case of influenza (infection and symptoms) (Jefferson *et al.*, 2012). (Note that these figures only account for reductions in illness rates amongst children participating in clinical trials, and do not measure the indirect herd protection inevitably offered to other unvaccinated children nor to adults. Since elderly adults are highly vulnerable, herd protection is likely to have an especially significant benefit for this group. We will return to this point below.) A systematic review of 14 RCTs conducted in 2005 suggests the VE of the influenza vaccine for children aged greater than 2 years is 79 per cent (Jefferson *et al.*, 2005). Other analyses suggest that VE can be as high as 87 per cent if children are vaccinated in consecutive years (Rhorer *et al.*, 2009).

There are two influenza vaccines currently available for children: the live attenuated influenza vaccine (LAIV) and the trivalent inactivated vaccine (TIV). Some evidence suggests that the LAIV can be more efficacious than the TIV and can be administered by nasal spray (as opposed to traditional percutaneous injection) (Jefferson *et al.*, 2012). This makes it easier to administer in group-based vaccination settings and more acceptable for needle-phobic children (and parents). We note that recently the CDC stopped recommending LAIV vaccination for children because of concerns about vaccine effectiveness based on a US study in 2015/2016 season (CDC, 2016). However, studies in the UK, Finland, Canada, as well as in the USA showed vaccine impact with the same vaccine in that season (Chambers *et al.*, 2016; Pebody *et al.*, 2016; Nohynek *et al.*, 2016). The UK is now the only country with a large childhood LAIV programme, and recent data continue to show a substantial impact of the vaccine on influenza in children and the wider population

Evidence shows that vaccinating children against influenza may significantly decrease school absenteeism for vaccinated children and their siblings (King *et al.*, 2006), while other studies suggest that vaccinating children against influenza may significantly decrease illness rates in, and work absenteeism of, parents as well (King *et al.*, 2006).

### What Are the Health Risks to Vaccinated Individuals?

Meta-analyses have combined research efforts assessing over 250,000 children under the age of 18 years. These have found no evidence that vaccinating children against influenza results in any significant risk of developing clinically important adverse events. The risks of a child developing a serious complication from the influenza vaccine are therefore thought to be less than 1 in every 250,000 cases (France *et al.*, 2004).

Observational assessment in Japan during the period of its mandatory schoolchildren influenza vaccination programme suggests the risks of ‘significant, severe side effects’ from the influenza vaccine amongst children to be less than 1 out of every 5 million cases (Reichert, 2002). Though the precise definition of these ‘significant’ side effects has not been adequately defined in the literature, the American Food and Drug Administration (FDA) suggests a ‘serious’ side effect is one that is either life-threatening or requires hospitalization, where other ‘important medical events’ include the development of seizures (without the need for hospitalization) or the administration of treatment in an emergency setting (U.S. Food and Drug Administration, 2016).

Though the case-fatality rate for the most severe form of vaccine-related allergy (e.g. anaphylaxis) has not been clearly defined (largely due to inconsistencies in case definitions), some have shown the case fatality rate for anaphylaxis from any cause to be between 0.7 and 20 per cent (Triggiani *et al.*, 2008). This indicates that the case-fatality rate for the influenza vaccine for children is also very low and likely to be less than 1 in every 25 million.

There is no evidence specific to children that influenza vaccination increases the risk of developing GBS, though some may consider the association in adults as weak evidence that similar pathology may develop in children. A recent retrospective study assessed 8.5 million paediatric vaccine recipients and concluded that there was no evidence of any increased risk of developing GBS from vaccines of any kind, including influenza (Baxter *et al.*, 2013).

### Overall Risk–Benefit Profile for Vaccinated Children

It has been estimated that in the 2015/2016 season 85 children died of influenza-related disease in the USA (CDC FluView, 2016). Although we could not find data on how many of these children were unvaccinated, there is no reason to think that the proportion has significantly changed in recent years. This seems supported by a CDC statement in a 2013 report, according to which ‘[t]he proportions of paediatric deaths occurring in children who were unvaccinated and those who had high-risk conditions are consistent with what has been seen in previous seasons’ (CDC, 2013a). Thus, for example, we know that during the 2012/2013 season at least 105 children died from influenza-related disease in the USA, that around 90 per cent of these deaths were in children unvaccinated against influenza and that around 40 per cent were in children with no recognized health problems (CDC, 2013a). Thus, we can estimate that around 38 otherwise healthy, unvaccinated US children died from the flu in the 2012/2013 influenza season.

The US Census Bureau suggests the population of US children aged under 18 years in 2010 was around 74 million (U.S. Census Bureau, 2011), and we can assume this datum did not significantly change in 2013. Assuming the population of *all* healthy children (under 18 years of age) in the USA was also 74 million (which is certainly an overestimate), CDC estimates (CDC, 2016a), according to which 56 per cent of children were vaccinated against influenza in 2012/2013, imply that about 41 million of these children were vaccinated against influenza and 33 million were unvaccinated. Thus, the estimated 38 influenza-related deaths amongst the 33 million healthy, unvaccinated children in the USA in 2012/2013 translate to an influenza mortality rate for unvaccinated children of greater than 1 per million.

Because the risk of death posed by electing not to vaccinate a child against influenza (more than 1 per million) is greater than estimates of the risk posed by vaccination (1 per 25 million), and because death is the most significant negative outcome of influenza, vaccinating children against influenza is clearly expected to be in a child’s best interests.

### What Are the Benefits for the Elderly and Other Vulnerable Citizens?

The majority of evidence correlating increased influenza vaccination uptake in children with decreases in influenza-associated mortality comes from probabilistic mathematical models. This is because the benefits of herd protection against influenza are difficult (and expensive) to measure in large, well-designed RCTs.

Models suggest that vaccinating 20 per cent of school-aged children can decrease adult mortality more than vaccinating 90 per cent of those aged over 65 years (Longini, 2012). With vaccination coverage of 40 per cent, serious morbidity and mortality could be reduced by 70 per cent in the elderly. As vaccination rates increase, incremental benefits would likely be observed with upper estimates that 70 per cent coverage amongst children could prevent 100 million cases of influenza in the USA every year (Basta *et al.*, 2009). Some pro-vaccination programmes might be capable of achieving coverage rates in excess of 90 per cent; and if similar coverage is attained for children against influenza, attack rates could decrease by two-thirds in children and by nearly 80 per cent in older adults (Reichert, 2002). The resultant reductions in influenza-associated deaths amongst elderly populations would be profound.

Though it is difficult to extrapolate the results of these models into recognizable reductions in total influenza-related mortality (e.g. either by registered respiratory and cardiovascular deaths or other ‘all-cause’ mortality estimates), there is important ecological data that gives further insight. Researchers have retrospectively analysed a universal schoolchildren influenza vaccination programme implemented in Japan from 1977 to 1987 and found that all-cause mortality decreased by *at least* 37,000 deaths (mainly in the elderly) for each year of the programme's enforcement, resulting in a two-thirds reduction in total influenza-related deaths. The programme ended because of unfounded public fear about its safety and effectiveness (Reichert *et al.*, 2001). This two-thirds (or 66 per cent) reduction in all-cause deaths would therefore be considered ‘great’ (e.g. greater than 50 per cent) according to our criteria for assessing effects on decreasing total influenza-related mortality.

Ecological evidence from the USA supports similar (albeit less dramatic) conclusions. In 1968 vaccination of 85 per cent of school children in Tecumseh, Michigan corresponded with a 67 per cent community-wide decrease in influenza-like attack rates (Monto *et al.*, 1970). More recently, increases in vaccination uptake in Texas to rates between 20 and 25 per cent have been shown to decrease medically attended acute respiratory illness by up to 18 per cent amongst adults (Glezen, 2006).

## Part 2—Beyond Effectiveness

If our aim is to achieve the greatest reduction in influenza- and influenza vaccine-related morbidity and mortality, vaccinating children against influenza seems the obvious strategy. Unlike vaccinating the elderly and/or HCP, vaccinating children may limit the transmission of influenza to vulnerable citizens living in the community and offer them indirect herd protection against harmful influenza infections. The potential benefits from vaccinating children are the greatest—as referenced, data from Japan suggest decreases in all-cause mortality of as much as two-thirds (Reichert *et al.*, 2001)—and the risks are minimal.

However, basing influenza prevention purely on such considerations does not account for that fact that vaccination strategies may have different moral costs.

There are at least three respects in which child-focused pro-vaccination strategies might plausibly be thought to pose greater moral costs than HCP- or elderly focused strategies. First, they might be thought to be more threatening to autonomy, since children cannot consent to vaccination. Second, they might be thought to problematically target a vulnerable group. And third, they might be thought to use children as a means to benefit others.

We will argue, however, that none of these considerations counts decisively against child-focused strategies.

Consider first the worry regarding autonomy. It is true that children are not able to consent to vaccination and that vaccination is thus always effectively mandatory from the perspective of the child, even if it is voluntary from the perspective of parents; the point is that the person who receives the vaccination—the child—cannot validly consent to it. In contrast, HCP- and elderly focused pro-vaccination strategies can be designed in such a way that the person who receives the vaccination consents to it. However, it does not follow that child-focused strategies undermine the autonomy of those whom they target more than do HCP- or elderly focused strategies. It is plausible that children are not autonomous and thus have no autonomy to be threatened. Indeed, their lack of autonomy may be precisely what explains why they cannot validly consent to vaccination, in the same way as they often cannot validly consent to receiving medical treatments or to any other intervention for which parents’ consent is required. For this reason, it is normally thought that decisions regarding medical interventions for children should be made solely or primarily on the basis of what is in the child’s best interests. We have argued above that flu vaccination typically is in a child’s best interests—that is, the benefits can be expected to outweigh the expected harms. This is true, despite the fact that most non-vaccinated children will not acquire influenza in a given season; the expected benefits of vaccination in the form of reduced influenza morbidity and mortality are small, but nevertheless greater than the expected harms from the vaccination.

It might be objected that it is the autonomy of parents, not that of children, that is threatened by child-focused pro-vaccination strategies. If these strategies make it impossible or difficult for parents to opt their children out of vaccination, parents will have lost some control over the manner in which their children are raised. Some hold that, for this reason, when mandating interventions against parental wishes, we apply a more stringent best interests standard than simply ‘the intervention has greater expected benefits than expected costs’. For instance, Diekema (2004) defends a standard according to which the intervention should be mandated only if there is a significant risk of serious preventable harm in the absence of the intervention and argues that childhood vaccination is unlikely to meet this standard.

We have two responses to this objection.

First, it applies only to pro-vaccination strategies that mandate vaccinations against parental wishes. As noted above, we do not suppose that child-focused strategies would necessarily be mandatory in this sense.

Secondly, even if Diekema’s standard is appropriate for mandatory medical interventions with no significant third-party effects, it might not be appropriate for mandatory vaccinations, where there is the potential for significant effects on others. In the case of vaccination, parental autonomy must be balanced not only against the best interests of the child but *also* against the health of third parties, given that non-vaccination poses a risk of harm to third parties, and these harms can create moral reasons in favour of vaccination (Dawson 2007, Navin 2013). If mandating childhood influenza vaccination against parental wishes would prevent substantial expected harms to third parties, reasons to avert those harms might alone offset reasons to respect parental autonomy. In that case, it might not be necessary, to justify mandatory vaccination, that the vaccinated children also receive a substantial benefit.^2^ For this reason, we remain open to the justifiability of mandatory childhood influenza vaccination programmes.

Consider next the worry regarding vulnerability. It might be thought problematic that child-focused vaccination strategies impose risks on a vulnerable group: children. It is doubtful whether this distinguishes child-focused strategies from elderly focused ones, since, at least in terms of their physical well-being, the elderly are arguably equally vulnerable. More importantly, however, this worry is undermined by the fact that, though childhood influenza vaccination may be attractive primarily because of its benefits for the elderly, it is typically, as we have just emphasized, also to the benefit of the vaccinated child. The vulnerability of children is ethically important because it suggests that others ought to take special care to protect their interests, but given the empirical data cited above, it is plausible that the best way to protect the interests of children against influenza- and influenza vaccine-related morbidity and mortality is precisely to promote childhood influenza vaccination.

Finally, consider the concern that child-focused vaccination strategies use children as a means to benefitting the elderly. The ‘Formula of Humanity’ variant of Kant’s Categorical Imperative requires that rational beings are never treated merely as a means to an end but always also as ends in themselves (Kant 1785/1996, esp. p. 429). The idea here is that everyone should be treated as having an intrinsic value of their own, rather than being merely a tool useful for progressing the goals of others. On a strict interpretation, this view rules out treating a person in a way that sets back her interests to advance the interests of others, unless, or perhaps even if, this is done with that person’s consent. It could be argued that influenza vaccination of children would violate this requirement by imposing risks on children without their consent to prevent others (for example, the elderly) from suffering the negative consequences of influenza.

It is highly doubtful whether the strict interpretation of the Kantian requirement specified above is correct. It is widely accepted that it is sometimes morally permissible to nonconsensually set back one person’s interests to protect or benefit others. Indeed, this is even accepted within medicine, and especially within public health. Many would hold, for example, that public health authorities may justifiably impose quarantine or mandated treatment in the context of a pandemic.

However, even if we should accept the strict Kantian requirement, it will not rule out child-focused pro-vaccination strategies. As noted above, influenza vaccination can be expected to confer net benefits on children. Though it poses some risks, we demonstrated above that the benefits are typically greater. Thus, even if child-focused pro-vaccination strategies are motivated mainly by a concern to protect the elderly, and even if their main benefits accrue to the elderly, they cannot aptly be characterized as setting back the interests of children to advance the interests of others. They advance both the interests of children and those of others. It is permissible in Kantian terms to use a person as a means, provided that the person is also treated as an end. Given that vaccination reduces a child's risk of death and morbidity, it is acceptable that vaccinating children is also a means to creating herd immunity to protect the elderly.

## Part 3—The Practicalities of Pro-vaccination Programmes

We have argued that increasing vaccination uptake amongst children could have profound impacts on decreasing influenza-related morbidity and mortality. We have explored some of the potential moral costs for strategies focused on vaccinating children, and argued that these do not weigh strongly (if at all) for the case of vaccinating children against influenza. We therefore believe there is a strong theoretical case for targeting influenza vaccination programmes at children.

In terms of the practicalities of increasing vaccine uptake, the UK has already proven that it is possible to roll out a schools-based programme, both in terms of public health infrastructure and financial cost. From a cost–benefit perspective, studies suggest savings of around US$35 per vaccinated child in group-based vaccination programmes (White *et al.*, 1999), so school-based vaccination programmes are likely to be cost-saving. We acknowledge that other countries might not have the money or public health infrastructure to set up similar programmes, even despite the cost savings they might expect in the long term, which is an obvious barrier for any vaccination programme, including those targeting children.

Because the influenza vaccine needs to be given annually, as opposed to other childhood vaccines like measles which is given twice only during childhood, we suspect school-based vaccination programmes may be most ideal at increasing uptake amongst school-aged children. Given potential increases in anti-vaccine trends, some may be concerned about how including another vaccine for school-aged children may affect public and parental trust of vaccines. Honest information sharing between schools, the public health sector delivering the vaccines, and parents would obviously be necessary and important, the same for the administration of any other vaccine.

We suggest school-based vaccination programmes, where the influenza vaccine is offered for free during school hours, might be an effective strategy for increasing vaccination uptake amongst school-aged children in countries where this is feasible. Looking at vaccine uptake rates in the UK since implementing school-based influenza vaccination for free, where vaccine uptake amongst school age children has not exceeded 63 per cent (England, 2017), further consideration of other pro-vaccination strategies might become increasingly relevant in future, such as introducing an ‘opt out’ approach, for example.

## Conclusion

Coming years will likely see increases in globalization, population density and ageing of populations in industrialized nations, prompting a need for urgent re-evaluation of current influenza prevention strategies (Glezen, 2006). Achieving herd immunity is the most effective way of decreasing the detrimental medical, social and financial consequences of seasonal influenza epidemics. We have argued that this can be most efficiently achieved through vaccination strategies that target children. We have also argued that the moral costs of such strategies do not outweigh their benefits, and that it might be cost-effective for countries to implement pro-vaccination programmes targeting children who can afford it and have the public health infrastructure to support such programmes.

## Funding

This work was supported by Wellcome Trust [grant number 100705/Z/12/Z to T.D.]; Uehiro Foundation on Ethics and Education [to T.D.]; Oxford Martin Programme on Collective Responsibility for Infectious Disease [to T.D., J.S. and A.G.]; and A.J.P. was supported by the NIHR Oxford Biomedical Research Centre and the Oxford Martin School.

## Footnotes

1. Differences in case-fatality rates may be higher in less developed countries where access to supportive intensive medical care (e.g. non-invasive or invasive respiratory support) might not exist or be available.

2. For similar claims, more fully developed, see Verweij and Dawson (2004: 3125), Flanigan (2014) and Pierik (2016).

3. For those offered free vaccination via the ‘school-based’ vaccination programme for children aged between 2 and 11 years in the UK.

4. An average for all US children aged between 6 months and 17 years.
